# Chronic photodistributed hyperpigmented patches in a middle-aged man

**DOI:** 10.1016/j.jdcr.2025.08.031

**Published:** 2025-09-18

**Authors:** Julian Cortes, Michael Haft, Jason Seidman, Taraneh Paravar

**Affiliations:** Department of Dermatology, University of California, San Diego Health, San Diego, California

**Keywords:** kratom, kratom induced hyperpigmentation, mytragina, supplement induced hyperpigmentation

## Case description

**Fig 1:**
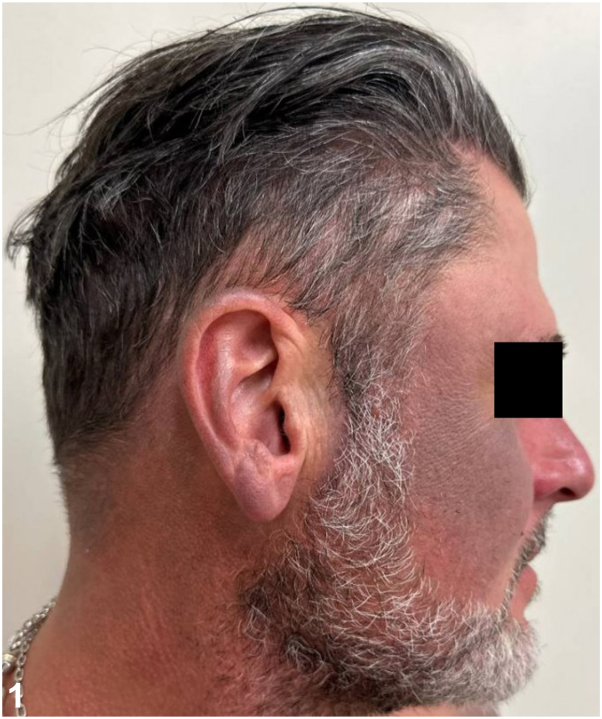

**Fig 2:**
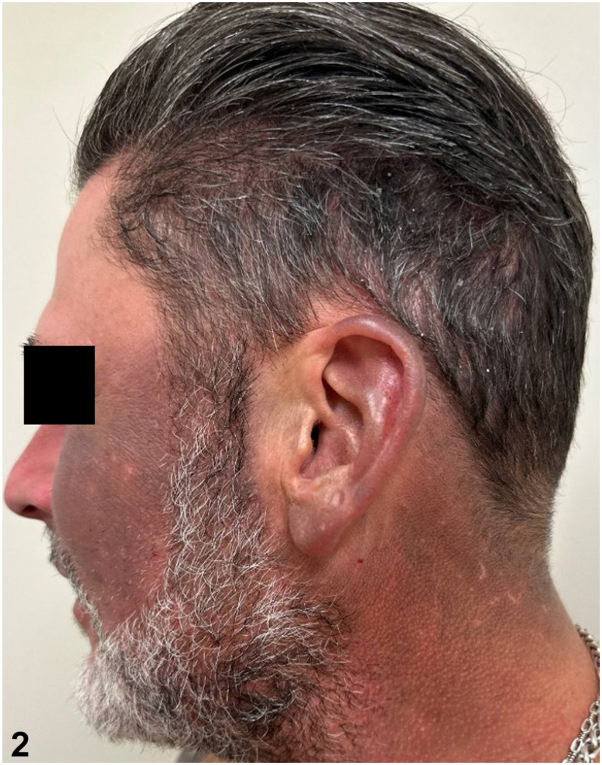

**Fig 3:**
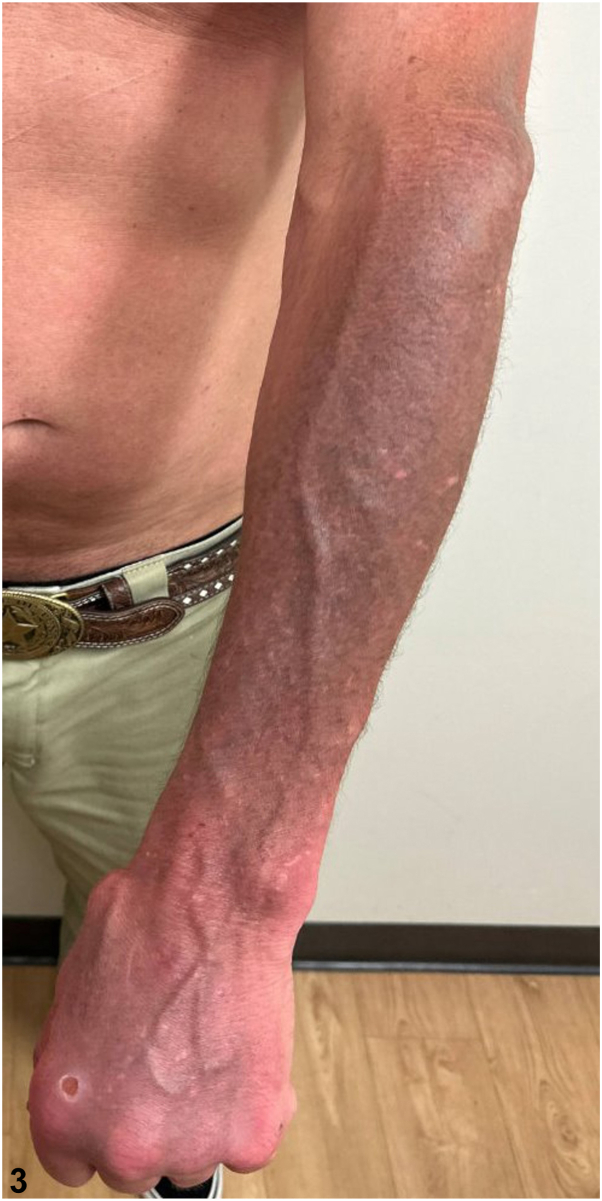

A 49-year-old man with a history of psoriasis, osteoarthritis, anxiety, depression, and polysubstance abuse presented to the dermatology clinic with a 3-year history of progressive darkening of the skin on the face, neck, and arms. He reported frequent sun exposure with inconsistent sunscreen use, contact with hydraulic oil, as well as daily kratom consumption over 8 years for anxiety management. After 6 months of sun avoidance, the patient noted no improvement in his skin discoloration. Physical examination revealed photodistributed blue-gray hyperpigmented patches involving the bilateral ears, temples, cheeks, mid-face, neck, and dorsal forearms (Fig 1, Fig 2, Fig 3). Punch biopsy from the affected skin demonstrated red-to-orange spherical pigment granule aggregates within both dermal melanophages and scattered freely in the perivascular and interstitial papillary dermis (Fig 4). These granules stained positively with Fontana-Masson, indicating melanin (Fig 5), and were negative on Prussian blue staining, ruling out hemosiderin.

**Figure:**
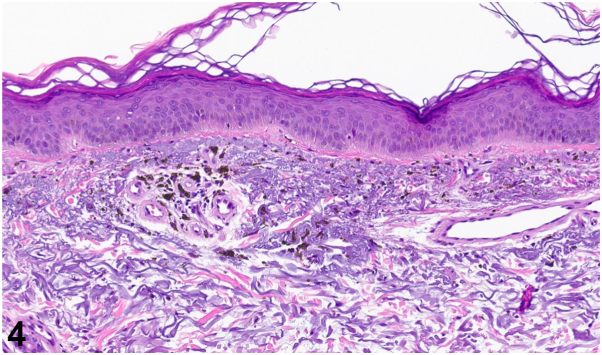

**Figure:**
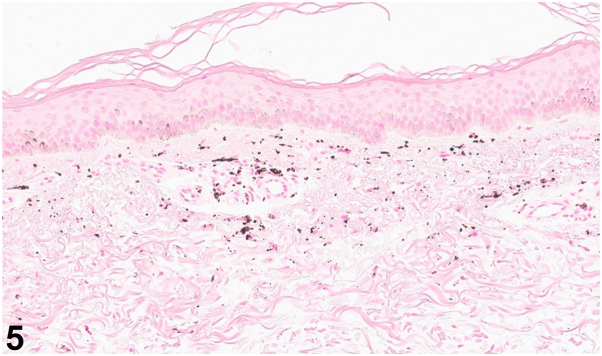

**Question and Answers: Which of the following is the most likely diagnosis?**

A. Hemosiderosis – Incorrect. Hemosiderosis, typically associated with chronic venous insufficiency, most often presents with hyperpigmentation of the distal lower extremities rather than the face and upper extremities. Additionally, the biopsy was negative for Prussian blue staining, suggesting an absence of dermal hemosiderin deposition and making this diagnosis less likely.
B. Postinflammatory hyperpigmentation – Incorrect. Postinflammatory hyperpigmentation typically follows visible skin inflammation and presents with brown patches most often due to increased epidermal melanin. This patient did not report a prior history of cutaneous injury and the dermal location of melanin granules within blue-gray pigmented patches is more consistent with drug-induced pigmentation rather than postinflammatory hyperpigmentation.
C. Lichen planus pigmentosus – Incorrect. Although lichen planus pigmentosus may present with similar clinical features, the absence of lichenoid interface dermatitis or band-like lymphocytic infiltrate on histopathology argues against this diagnosis.
D. Kratom-induced hyperpigmentation – Correct. The patient’s history of long-term kratom use, photodistributed blue-gray pigmentation, and histopathology showing Fontana-Masson–positive melanin granules within dermal melanophages support a diagnosis of kratom-induced hyperpigmentation. Negative Prussian blue staining further rules out hemosiderin as a cause of pigmentation.
E. Erythema dyschromicum perstans – Incorrect. Like lichen planus pigmentosus, erythema dyschromicum perstans may present similarly to kratom-induced hyperpigmentation. However, additional histologic findings, such as basal vacuolization or perivascular inflammatory infiltrate, would be expected.

**Which of the following best characterizes the clinical presentation and histopathologic features of kratom-induced hyperpigmentation?**

A. Depigmented patches with absent epidermal melanin and loss of melanocytes. – Incorrect. These features are consistent with vitiligo.
B. Blue-gray pigmentation on sun-exposed areas with pigment incontinence (Fontana-Masson positive) and minimal inflammation. – Correct.
C. Gray-brown patches in sun exposed areas with basal vacuolization, apoptotic keratinocytes, and a band like lymphocytic dermal infiltrate. – Incorrect. These features are consistent with lichen planus pigmentosus.
D. Blue gray discoloration of the face with yellow-brown banana shaped fibers in the dermis. – Incorrect. These features are consistent with ochronosis.
E. Blue black pigmented scars with dermal pigment (Perl’s Prussian Blue positive). – Incorrect. These features are consistent with minocycline-induced pigmentation.

## Discussion

Kratom is a herbal supplement whose active compounds, mitragynine and 7-hydroxymitragynine, exhibit strong affinity for opioid receptors. As such, kratom has been used for self-treatment of chronic pain, anxiety, and depression, and has become increasingly prevalent within the United States with an estimated 1.7 million users in 2021.^1^ Cases published in the literature have reported a photodistributed blue-gray hyperpigmentation arising years after chronic kratom use, with most cases demonstrating superficial dermal melanin pigment deposition with positive Fontana-Masson staining and minimal presence of inflammatory cells.^2^, ^3^, ^4^, ^5^

The mechanism by which chronic kratom use might lead to hyperpigmentation is not entirely clear. One proposed mechanism involves activation of μ-opioid receptors by endogenous β-endorphin, which may stimulate melanogenesis in epidermal melanocytes. By mimicking β-endorphin, mitragynine and 7-hydroxymitragynine may produce similar hyperpigmentation. Additionally, sunlight exposure may trigger free radical generation from kratom within the epidermis. Subsequent DNA damage may result in the release of pigmentary mediators in a photodistributed fashion.^3^ Kratom may also antagonize dopamine receptors, potentially promoting production of melanocyte-stimulating factors within the pituitary gland.^4^ Further research is needed to assess the role kratom and other opioid agonists may play in the development of this photodistributed hyperpigmentation.

Although cases of kratom-induced hyperpigmentation are increasingly reported, most attempts at treatment – including prolonged abstinence, sun avoidance, and topical lightening agents—have proven unsuccessful.^2^ Most recently, however, a case series demonstrated promising outcomes for 2 patients with use of the 730 nm picosecond titanium-sapphire laser.^5^ As dermatologists pursue potential treatment modalities for kratom-induced hyperpigmentation, appropriate and individualized counseling of associated risks and benefits should be provided for each patient. Given the potential withdrawal and psychoactive effects associated with discontinuing chronic kratom use, patients may require additional social or pharmacological support to achieve complete abstinence.

As kratom use continues to rise within the United States, dermatologists should be prepared to recognize the potential consequences its use may have on the skin and participate in shared decision making to identify appropriate individualized treatment courses.

## Conflicts of interest

Dr Haft has served as a sub-investigator on industry-sponsored clinical trials involving investigational therapies from the following pharmaceutical companies: AbbVie, Arcutis Biotherapeutics, Bausch Health, Castle Biosciences, Dermavant Sciences, Eli Lilly and Company, Galderma S.A., Janssen Pharmaceuticals, Regeneron Pharmaceuticals, and TARGET PharmaSolutions. Drs Seidman, Paravar, and Author Cortes have no conflicts of interest to declare.
